# CD4 nadir and neurocognitive trajectories in people living with HIV

**DOI:** 10.1007/s13365-024-01217-8

**Published:** 2024-06-10

**Authors:** Razmig Garabet, Will Dampier, Shinika Tillman, Kim Malone, Zsofia Szep, Amy Althoff, Vanessa Pirrone, Michael R. Nonnemacher, Brian Wigdahl, Maria Schultheis, Kathryn N. Devlin

**Affiliations:** 1 Department of Microbiology and Immunology, Drexel University College of Medicine, Philadelphia, PA USA; 2 Center for Molecular Virology and Translational Neuroscience, Institute for Molecular Medicine and Infectious Disease, Drexel University College of Medicine, Philadelphia, PA USA; 3 Department of Medicine, Division of Infectious Diseases and HIV Medicine, Drexel University, Philadelphia, PA USA; 4 Division of Infectious Diseases, Department of Medicine, University of Pennsylvania, Philadelphia, PA USA; 5 Sidney Kimmel Cancer Center, Thomas Jefferson University, Philadelphia, PA USA; 6 Department of Psychological and Brain Sciences, Drexel University, Philadelphia, PA USA

**Keywords:** HIV, HAND, Neurocognition, Longitudinal, CD4 nadir

## Abstract

Human immunodeficiency virus-associated neurocognitive disorders persist in the combination antiretroviral therapy era. CD4 nadir is a well-established predictor of cognition cross-sectionally, but its impact on longitudinal neurocognitive (NC) trajectories is unclear. The few studies on this topic examined trajectories of global cognition, rather than specific NC domains. The current study examined CD4 nadir in relation to domain-specific NC decline. 132 HIV + adults from the Temple/Drexel Comprehensive NeuroHIV Center, Clinical and Translational Research Support Core Cohort were administered comprehensive NC assessments longitudinally, with last visit occurring an average of 12 years after CD4 nadir. Linear mixed models were used to examine CD4 nadir in relation to longitudinal NC trajectories in three empirically identified NC domains: speed/executive function (S/EF), visuospatial memory (VM), and verbal fluency (VF). CD4 nadir was associated with change in VF (p = 0.020), but not with S/EF or VM. Specifically, those with CD4 nadir < 200 demonstrated increasing VF over time (p = .002), whereas those with CD4 nadir > 200 demonstrated stable VF (p = .568), though these differing trajectories may partly reflect regression to the mean or differential practice effect. CD4 dynamics over time were analyzed as potential mechanisms for the identified associations, with mixed findings. While low CD4 nadir has been associated with weaker neurocognition among people living with HIV, the results of this study suggest that low CD4 nadir is not associated with ongoing decline a decade later. Nadir-related deficits in VF may be stable or even improve over time, possibly reflecting the beneficial cognitive effects of long-term treatment and immune reconstitution.

**Supplementary Information:**

The online version contains supplementary material available at 10.1007/s13365-024-01217-8.

## Introduction

The innovation of combination antiretroviral therapy (cART) has markedly reduced mortality rates among people living with HIV-1 (PLWH; Hernán 2010). However, HIV-associated neurocognitive disorders (HAND) persist globally during the cART era (Wang et al. 2020; Wei et al. 2020). These neurocognitive disorders' trajectories are heterogenous, with a mix of stable, worsening, and improving courses (Heaton et al. 2015; Gott et al. 2017). Thus, there is an ongoing need to identify risk factors for cognitive decline.

Low CD4 nadir is a predictor of more severe neurocognitive deficits (Heaton et al. 2010; Ellis et al. 2011). It has also been associated with neuroanatomical changes, such as increased cortical thinning (Hassanzadeh-Behbahani et al. 2020) and ventricular expansion (Cohen et al. 2010). Although the predictive value of CD4 nadir with respect to neurocognitive deficits has been well established cross-sectionally, its impact on longitudinal cognitive trajectories is unclear. Assessment of cognitive decline over time in relation to CD4 nadir has demonstrated conflicting results (Heaton et al. 2015; Naveed et al. 2022; Ellis et al. 2022). This variation in results could potentially be due to the methodology of assessment, as neurocognition was examined on a global scale, rather than in individual neurocognitive domains.

The neurocognitive domains most susceptible to HIV are wide-ranging and include executive function, motor skills, processing speed, episodic learning and retrieval, working memory, and verbal fluency (Woods et al. 2009). Furthermore, the neurocognitive profile of HAND is heterogeneous across individuals (Reger et al. 2002). Assessing cognition by domains can capture patterns of impairment that would be missed when assessing a global deficit (Dawes and Grant 2007). Because various aspects of HIV disease may differentially affect particular brain regions and neural systems, this approach can also elucidate relationships between specific risk factors and specific domains of cognitive decline. This emphasizes the importance of investigating domain-specific neurocognition when studying risk factors for developing HIV-associated neurocognitive decline.

There are several potential trajectories of cognitive impairments associated with low CD4 nadir. These impairments may resolve with time and immune reconstitution (Pfefferbaum et al. 2014). Alternatively, low CD4 nadir may have a persistent, albeit stable, influence on the level of cognitive impairment (Heaton et al. 2010; Ellis et al. 2011). Finally, low CD4 nadir could be associated with ongoing neuropathological cascades in the CNS viral compartment that lead to continued cognitive decline, even in the setting of good peripheral viral suppression (Ellis et al. 2022) Moreover, the relationship between CD4 nadir and cognitive trajectories may vary by cognitive domain, as neural systems may differ in their susceptibility to decline and their capacity to recover.

Because CD4 counts are historically measured in routine HIV care, CD4 nadir is an accessible, cost effective, and low-risk measurement of immune status and HIV disease history. Thus, finding a potential relationship between CD4 nadir and HAND trajectories can provide both patients and clinicians with an understanding of disease progression without the need for costlier, less accessible biomarker tests or more invasive procedures, such as lumbar puncture. Focusing on HIV-induced immune dynamics that may influence neurocognitive trajectories is imperative to enhance the understanding of disease progression, immune reconstitution, and ultimately patient care.

To our knowledge, no previous studies have examined the relationship of CD4 nadir to longitudinal domain-specific neurocognitive change. The current study examined the impact of CD4 nadir levels on trajectories of specific neurocognitive domains, as well as global neurocognitive trajectory, in PLWH.

## Methods

### Procedures

The current study used a longitudinal observational design. Participants were a subset of the Clinical and Translational Research Support Core (CTRSC) cohort of the Drexel University/Temple University Comprehensive NeuroHIV Center (CNHC) in Philadelphia, Pennsylvania. The CNHC CTRSC sample is a longitudinal cohort of adult PLWH, recruited by clinician referrals, word-of-mouth, flyers, and web-postings. CNHC cohort participants were eligible for the present study if they spoke English as a primary language and completed at least one neurocognitive assessment. The study was approved by the Drexel University College of Medicine institutional review board (IRB protocol 1609004807; Brian Wigdahl PI), and all procedures were conducted in agreement with its ethical standards. Participants completed written informed consent and were compensated for their involvement.

Each participant underwent a comprehensive examination, approximately annually, that included a medical interview, record review, and neurocognitive assessment. Nadir CD4 T cell levels were obtained through medical records, and through self-report when medical records were unavailable. Neurocognition was assessed with a comprehensive battery that included tests of fine motor speed and dexterity [Grooved Pegboard Dominant and Non-dominant hands (Kløve 1963)], psychomotor speed [Trail Making Test Part A (Reitan 1992)], executive function [Trail Making Test Part B (Reitan 1992)], verbal fluency [Category Fluency (Animals) and Letter Fluency (FAS; Benton et al. 1994)], and visuospatial episodic memory [Brief Visuospatial Memory Test-Revised (BVMT-R) Immediate Recall, Delayed Recall, and Recognition (Benedict 1997)]. Raw neurocognitive scores were converted to Z-scores using published normative adjustments for age, gender, education, and race/ethnicity (Heaton et al. 2004; Schretlen et al. 2010).

### Statistical analysis

Statistical analyses were conducted using IBM SPSS 28. Demographic and clinical differences between participants with one versus multiple visits, and with low versus high CD4 nadir, were examined using analysis of variance (ANOVA) and chi-squared or Fisher exact tests. Principal component analysis (PCA) with varimax rotation was utilized to determine specific neurocognitive domains in order to limit multiple comparisons. Based on PCA results, domain Z-scores were calculated by averaging the test Z-scores within each domain. Global cognition was derived by averaging Z-scores of every administered test. Two-step automatic cluster analysis (CA) of the resultant domain Z-scores was used to classify baseline global cognitive status. This allowed us to account for potential variability in trajectories related to baseline global cognitive status.

Longitudinal neurocognitive trajectories were analyzed by linear mixed modeling (LMM), with subject-specific random intercepts. Separate LMMs were conducted to model global cognition and each individual cognitive domain. The independent variables were CD4 nadir (high vs. low), time since nadir (years), baseline global cognitive status (impaired vs. unimpaired), and all two- and three-way interactions among them. CD4 nadir was dichotomized as high or low based on a cutoff of 200 cells/μl. A CD4 value below 200 cells/μl is a clinical hallmark for the diagnosis of acquired immunodeficiency syndrome (AIDS) and an indication of a more severe immunocompromise state (Garcia and Guzman 2023). The PCA and CA included baseline visits from all participants with at least one visit, while LMMs included only participants with multiple visits.

To aid in interpretation of whether the LMM findings reflected meaningful cognitive change, we computed reliable change indices (RCIs) using each participant’s baseline and last visit. We used the Maassen modified RCI method (Maassen 2004), a regression-based approach that offers advantages over other methods by accounting for measurement error (reliability), practice effects, initial position (i.e., regression to the mean), and inequality between test and retest variance (for a review of this and other RCI methods, see Hinton-Bayre 2010). Briefly, this method compares a participant’s follow-up score with a regression-based predicted score (based on that participant’s baseline score, and test and retest means and SDs from a normative sample), then divides this difference by a standard error term (based on test and retest SDs and test–retest reliability from a normative sample).

When computing RCIs, we applied normative data from Woods et al. (2006), which examined test and retest performance over a long interval (one year) in healthy control individuals using a nearly identical test battery to the present study; moreover, Woods et al. (2006) validated the RCIs resulting from these norms in a sample of PLWH. As these norms apply to raw scores from individual tests, to obtain a representative RCI for each domain, we computed RCIs using the raw score of the test with the highest factor loading in the PCA described above, or the second highest if the first was not available in the Woods et al. (2006) norms. Consistent with standard practice (Hinton-Bayre 2010), RCIs above 1.645 or below -1.645, representing change outside the 90% confidence interval, were categorized as representing clinically meaningful improvement or decline, respectively. Domain-specific RCI categories (improvement, stability, decline) were examined in relation to the number of repeated assessments and the interval between baseline and last visit using Kendall’s tau correlation; because these factors did not contribute to the computation of RCIs, this analysis was performed to ensure that they did not influence RCIs. Finally, domain-specific RCI categories were examined in relation to CD4 nadir group and/or baseline global cognitive status (if significantly associated in the LMMs) using chi-square tests or Fisher’s exact tests.

Changes in current CD4 T cell count were analyzed to investigate mechanistic explanations behind the primary findings: namely, whether CD4 nadir was related to changes in current CD4 count, and whether changes in CD4 count were in turn related to domain-specific cognitive changes. Binary groups of improving and stable/decreasing CD4 counts were determined by comparing CD4 counts at last visit versus baseline. Improving CD4 count was defined as an increase in CD4 count from less than 500 cells/μl to greater than 500 cells/μl. A 500 cells/μl cutoff was chosen because it is the clinical hallmark for transition from stage 1 to stage 2 HIV progression (Green et al. 2017) and because a cutoff of 200 cells/μl produced groups with insufficient sample sizes. A chi-squared test was then conducted to examine CD4 trajectories in relation to CD4 nadir counts. Finally, for any cognitive domains that showed an association with CD4 nadir in the LMMs, cognitive domain-specific RCI categories were further examined in relation to CD4 count trajectories using chi-squared tests or Fisher’s exact tests.

## Results

### Participant characteristics

Adult PLWH (301 in number) enrolled in the CNHC CTRSC cohort met inclusion criteria and completed at least one neurocognitive assessment. Of the 301 participants, 132 underwent multiple neurocognitive assessments and comprised the longitudinal sample for the study’s primary analysis. There were no significant differences in any demographic or HIV-related characteristics between participants with one versus multiple visits (Supplemental Table 1). However, there was a trend towards a between-group difference in latest viral load (p = 0.054), with a higher percentage of detectable viral load among people without follow-up visits. Of the 132 patients with at least two visits, 50 had at least three visits, and 14 had four visits, for a total of 328 visits. The average follow-up time was 4.9 years (SD = 2.1, range = 0.4–8.5) across all follow-up visits and 5.2 years (SD = 2.3) at last visit.

Participants ranged in age from 25–68 (M = 51.8), with a high school level of education on average (M = 11.8 years; Table 1). The sample was predominantly Black (92%), with somewhat more men (59%) than women. The participants were well treated, with 98% currently on cART, 91% with undetectable viral load, and clinically normal current CD4 levels (M = 686, SD = 302). Thirty eight percent of the participants had a CD4 nadir below 200. The average time since CD4 nadir at baseline was 6.5 years (SD = 3.8, range = 0.0–17.9) and the average time since CD4 nadir at the last visit was 12.4 years (SD = 6.1), with a maximum of 26.7 years. Individuals with CD4 nadir < 200 were less likely to be Black (p = 0.039) and had more years of education (p = 0.027), lower current CD4 counts (p < 0.001), and a longer time since their nadir (p < 0.001) than the CD4 nadir > 200 group.

**Table 1:** Demographics, HIV-related, and cognitive characteristics of the longitudinal sample

	**Total**	**Nadir < 200**	**Nadir > 200**	**p**
**N**	132	50 (38%)	82 (62%)	-
**Age**^a^	51.8 (7.6)	51.6 (6.9)	51.9 (8.0)	.782
**Education (years)**^a^	11.8 (2.3)	12.3 (2.5)	11.4 (2.1)	.027
**Gender**^b^	59% Men 41% Women	56% Men 44% Women	61% Men 39% Women	.589
**Race**^b^	92% Black 3.8% White 2.3% Hispanic 1.5% Other	88% Black 6.0% White 6.0% Hispanic 0.0% Other	95% Black 2.4% White 0.0% Hispanic 2.4% Other	.039
**Latest HIV Viral Load (< 200 copies/ml)**^b^	91%	86%	94%	.214
**On cART**^b^	98%	98%	98%	1.00
**Latest CD4 Count (cells/μl)**^a^	686 (302)	496 (267)	804 (261)	< .001
**Years Seropositive**^a^	18.3 (7.3)	18.4 (7.4)	18.2 (7.2)	.883
**Years on cART**^a^	15.7 (7.3)	16.5 (7.1)	15.2 (7.5)	.319
**Years Seropositive Before Starting cART**^a^	3.1 (5.0)	2.5 (3.8)	3.5 (5.6)	.253
**Years Since Nadir Date**^a^	6.5 (3.8)	8.0 (4.5)	5.7 (2.9)	< .001
**Impaired Global Cognitive Status**^b^	52%	57%	49%	.467

^a^Numerical variable. Values represent M (SD). Significance obtained from ANOVA

^b^Categorical variable. Significance obtained from Fisher’s exact test

### Defining cognitive domains and baseline cognitive status

In a PCA of baseline cognitive data from all participants with complete data in all 9 NC measures (n = 263), three components had eigenvalues greater than 1 and explained 43%, 15%, and 13% of the variance in cognitive test scores. Subsequent components each explained < 7.4% of the variance, and the scree plot revealed minimal added benefit after the third component. The three-factor solution had at least two items per factor, rotated factor loadings were strong (> 0.6), and each factor represented a distinct meaningful construct. The speed and executive function (S/EF) factor was comprised of Trail Making Test Parts A and B and Grooved Pegboard Dominant and Non-dominant Hands. Visuospatial memory (VM) was comprised of BVMT-R Immediate Recall, Delayed Recall, and Recognition. Verbal Fluency (VF) was comprised of Letter Fluency and Category Fluency (Supplemental Table 2). The rotated factors accounted for 29%, 24%, and 18% of variance, respectively, for a total of 71%.

A two-step CA was performed with participants with complete data in all three domain z-scores (n = 295). The CA revealed a two-cluster solution. Bayesian Information Criterion (BIC) values decreased markedly from the first to the second cluster, with diminishing reductions through the fifth cluster, and increased thereafter (Supplemental Table 3). The ratio of distance measures and the silhouette measure of cohesion and separation were highest for the two-cluster solution. The first cluster represented 54% of the sample (n = 158) and demonstrated impaired cognition [S/EF: M = -1.07 (SD = 0.67); VM: M = -1.49 (SD = 0.96); VF: M = -0.68 (SD = 0.79)], with scores approximately one SD below that of the second cluster (n = 137), which had intact cognition [S/EF: M = -0.06 (SD = 0.66); VM: M = -0.18 (SD = 0.74); VF: M = 0.55 (SD = 0.71)]. Baseline global cognitive status was not associated with CD4 nadir (Table 1) or with retention within the study (Supplemental Table 1).

### CD4 nadir and baseline cognitive status in relation to cognitive trajectories

In participants with multiple visits (n = 132), a LMM was initially used to investigate any relationship between CD4 nadir and global cognition over time (Table 2). A trend towards statistical significance was observed for the association between CD4 nadir (p = 0.071) and the trajectory of global cognitive functioning. Baseline global cognitive status was also associated with change in global cognition over time (p < 0.001). Significant main effects of baseline global cognitive status and time were also present (both p < 0.001).

**Table 2:** Fixed effects from linear mixed model for global cognition

	Est	SE	df	t	p
Intercept	-0.74	0.06	263	-13.02	< .001
**Global Cognitive Status**	**1.40**	**0.11**	**264**	**12.34**	** < .001**
CD4 Nadir	0.12	0.12	280	1.04	.297
**Years Since Nadir**	**0.02**	**0.01**	**281**	**-1.29**	** < .001**
Global Cognitive Status * CD4 Nadir	-0.31	0.24	281	-1.29	.198
Global Cognitive Status * Years Since Nadir	**-0.04**	**0.01**	**264**	**-4.77**	** < .001**
CD4 Nadir * Years Since Nadir	-0.02	0.01	279	-1.81	.071
Global Cognitive Status * CD4 Nadir * Years Since Nadir	0.01	0.02	280	0.72	.470

**Fig. 1:**
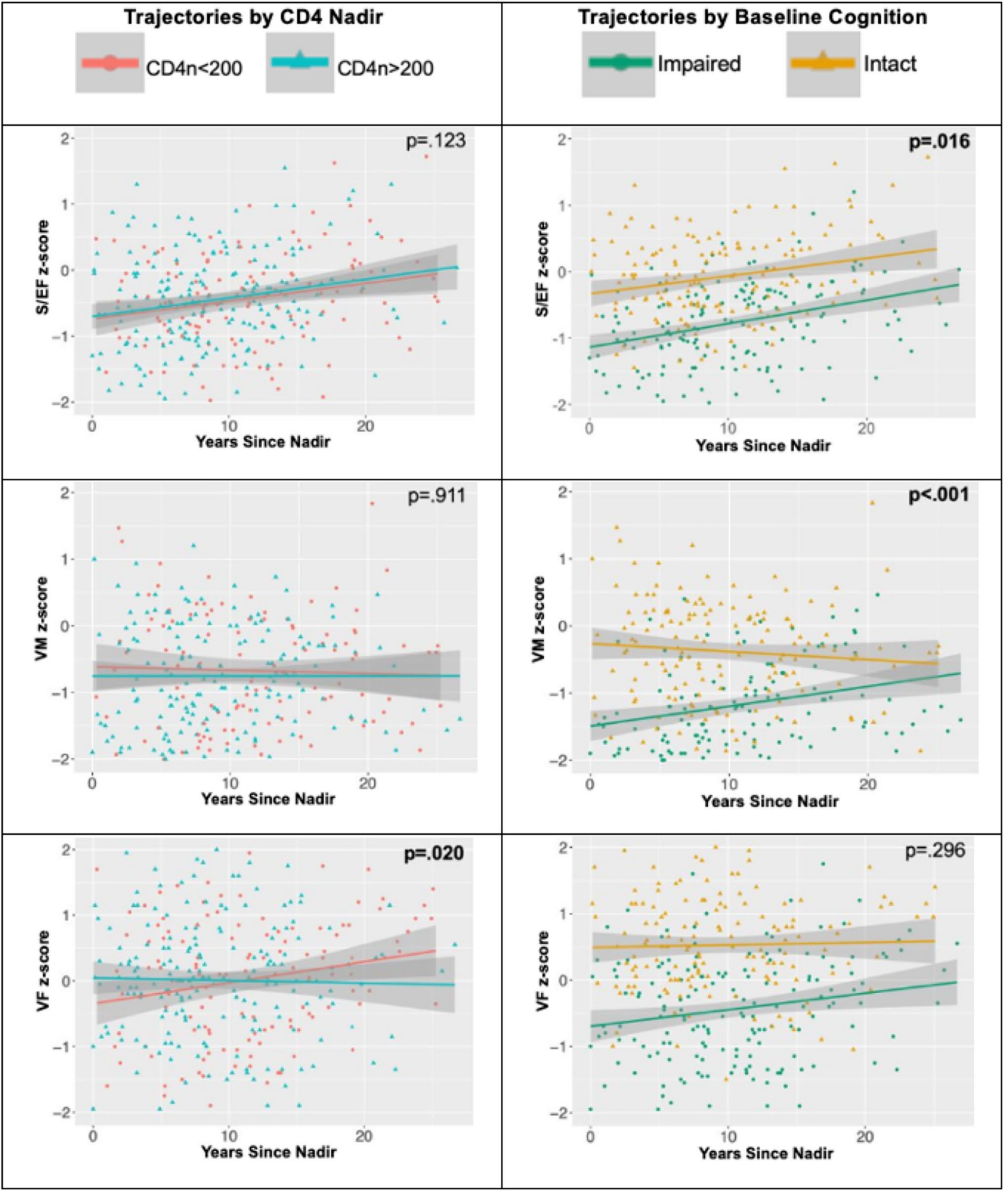
CD4 nadir was associated with the trajectory of VF, but not S/EF or VM. Baseline global cognitive status was associated with the trajectory of S/EF and VM, but not of VF. Graphs depict domain-specific cognitive trajectories by CD4 nadir (left) and baseline global cognitive status (right). P values represent the effect of CD4 nadir * time since nadir (left) or the effect of baseline global cognitive status * time since nadir (right). CD4n = CD4 nadir

To further explore the cognitive domains driving these findings, LMMs assessed the association between CD4 nadir and longitudinal trajectories of each cognitive domain (Fig. 1; Tables 3, 4 and 5). CD4 nadir was associated with change in VF over time (p = 0.020 for the interaction of CD4 nadir * years since nadir), but not with change in S/EF (p = 0.123) or VM (p = 0.911). Specifically, the CD4 nadir < 200 group improved in VF [+ 0.035 SD/year, SE = 0.01, t(121) = 3.09, p = 0.002], while the CD4 nadir > 200 group was stable [-0.006 SD/year, SE = 0.02, t(148) = -0.572, p = 0.568]. There was also a main effect of CD4 nadir, such that the CD4 nadir > 200 group had a significantly higher VF z-score than the CD4 nadir < 200 group when collapsing across time points (p = 0.050). The CD4 < 200 group initially began with a lower VF z-score and increased, eventually equaling that of the CD4 nadir > 200 group roughly a decade post-nadir, and then exceeded it.

**Table 3:** Fixed effects from linear mixed model for Speed/Executive Function

	Est	SE	df	t	p
Intercept	-0.79	0.08	261	-10.15	< .001
**Global Cognitive Status**	**1.07**	**0.16**	**262**	**6.86**	** < .001**
CD4 Nadir	0.22	0.16	286	1.33	.184
**Years Since Nadir**	**0.03**	**0.01**	**248**	**4.62**	** < .001**
Global Cognitive Status * CD4 Nadir	-0.42	0.33	287	-1.27	.205
Global Cognitive Status * Years Since Nadir	**-0.03**	**0.01**	**251**	**-2.43**	**.016**
CD4 Nadir * Years Since Nadir	-0.02	0.01	269	-1.55	.123
Global Cognitive Status * CD4 Nadir * Years Since Nadir	0.02	0.02	270	0.88	.382

**Table 4:** Fixed effects from linear mixed model for Visuospatial Memory

	Est	SE	df	t	p
Intercept	-1.12	0.10	268	-11.72	< .001
**Global Cognitive Status**	**1.87**	**0.19**	**269**	**9.75**	** < .001**
CD4 Nadir	-0.18	0.20	275	-0.90	.367
Years Since Nadir	0.01	0.01	289	1.64	.102
Global Cognitive Status * CD4 Nadir	-0.34	0.41	276	-0.84	.405
Global Cognitive Status * Years Since Nadir	**-0.08**	**0.02**	**290**	**-4.85**	** < .001**
CD4 Nadir * Years Since Nadir	0.002	0.02	297	0.11	.911
Global Cognitive Status * CD4 Nadir * Years Since Nadir	-0.001	0.03	297	-0.03	.971

**Table 5:** Fixed effects from linear mixed model for Verbal Fluency

	Est	SE	df	t	p
Intercept	-0.04	0.10	246	-0.46	.644
**Global Cognitive Status**	**1.28**	**0.19**	**246**	**6.67**	** < .001**
**CD4 Nadir**	**0.40**	**0.20**	**257**	**1.97**	**.050**
Years Since Nadir	0.01	0.01	186	1.07	.286
Global Cognitive Status * CD4 Nadir	0.01	0.40	257	0.04	.972
Global Cognitive Status * Years Since Nadir	-0.02	0.02	186	-1.05	.296
CD4 Nadir * Years Since Nadir	**-0.04**	**0.02**	**204**	**-2.34**	**.020**
Global Cognitive Status * CD4 Nadir * Years Since Nadir	0.02	0.03	203	0.55	.584

Baseline global cognitive status was associated with change in S/EF (p = 0.016 for the interaction of cognitive status * years since nadir) and VM (p < 0.001) over time, but not with VF change (p = 0.296). For S/EF, the sample overall improved over time (p = 0.006 for the main effect of years since nadir), but this effect differed by baseline global cognitive status. Namely, the baseline-impaired cognitive group improved in S/EF [+ 0.04 SD/year, SE = 0.01, t(143) = 5.62, p < 0.001], while the baseline-intact cognitive group was stable [+ 0.01 SD/year, SE = 0.01, t(138) = 1.40, p = 0.165]. For VM, the baseline-impaired cognitive group improved [+ 0.05 SD/year, SE = 0.01, t(122) = 4.62, p < 0.001], while the intact group declined subtly [-0.03 SD/year, SE = 0.01, t(144) = -2.35, p = 0.020]. Finally, there were significant main effects of baseline global cognitive status for all three domains (all p < 0.001), with better cognition across visits in those with intact baseline cognitive status.

### CD4 nadir and baseline cognitive status in relation to reliable cognitive change

To aid in clinical interpretation of cognitive trajectories, domain-specific RCIs were computed to adjust for factors such as practice effects and regression to the mean. RCI computations used the test with the highest PCA loading and available RCI norms (Woods et al. 2006; VF: Letter Fluency; S/EF: Grooved Pegboard Non-dominant; VM: BVMT-R Delayed Recall). In the overall sample, rates of reliable cognitive improvement and decline, respectively, were 9.4% and 13.3% for VF, 30.6% and 28.1% for S/EF, and 3.8% and 30.8% for VM. Thus, the net cognitive trajectory was stable in the VF and S/EF domains and declining in the VM domain. Domain-specific change categories were not related to inter-test interval (VF: τ = -0.08, p = 0.280; S/EF: τ = -0.04, p = 0.598; VM: τ = -0.05, p = 0.505) or number of repeated assessments (VF: τ = 0.10, p = 0.237; S/EF: τ = -0.01, p = 0.875; VM: τ = -0.06, p = 0.438).

Given LMM results, we examined reliable VF change in relation to CD4 nadir and reliable change in S/EF and VM in relation to baseline global cognitive status. VF improvement was slightly more common in the low nadir group (12.5%) than the high nadir group (7.5%; Fisher’s p = 0.364), while VF decline was slightly more common in the high nadir group (15.0%) than the low nadir group (10.4%; χ^2^ p = 0.459), but neither comparison was statistically significant. In line with LMM results, S/EF improvement was significantly more common in the baseline-impaired cognitive group (40%) than the baseline-intact group (22%; χ^2^ p = 0.034), while S/EF decline was similarly common in the two groups (baseline-impaired: 28.3%; baseline-intact: 28.8%, χ^2^ p = 0.954). Finally, the rate of VM improvement was 6% in the baseline-impaired cognitive group and 2% in the baseline-intact group (Fisher’s p = 0.369), while VM decline was significantly more common in the baseline-intact group (41.7%) than the baseline-impaired group (20.9%; χ^2^ p = 0.011).

### CD4 trajectories as a potential mechanism of VF change

Given the primary findings, CD4 dynamics over time were analyzed as a potential mechanism of the association between CD4 nadir and VF change. CD4 nadir was examined in relation to clinically meaningful change in current CD4 (i.e., improvement from < 500 to > 500 cells/μl vs non-improvement), which in turn was examined in relation to the RCI change category (i.e., improvement, stability, decline) for VF. There was a significant association between CD4 nadir and CD4 change, such that 24.0% (12/50) of the CD4 nadir < 200 group versus only 2.5% (2/81) of the CD4 nadir > 200 group demonstrated an improving CD4 trajectory, [x^2^(1, N = 131) = 15.02, p < 0.001]. Regarding CD4 change in relation to reliable VF change, 21.4% (3/14) of participants with improving CD4 trajectories demonstrated VF improvement, versus only 8.0% (9/113) of participants with unimproved CD4 trajectories, but this difference was not statistically significant [Exact p = 0.129]. Similarly, 7.1% (1/14) of participants with improving CD4 trajectories and 13.3% (15/113) of participants with unimproved CD4 trajectories demonstrated VF decline [Exact p = 1.00].

## Discussion

The purpose of this study was to examine the long-term outcomes of CD4 nadir on domain-specific neurocognitive trajectories in PLWH. A lower CD4 nadir count has been associated with decreased neurocognitive functioning cross-sectionally (Heaton et al. 2010; Ellis et al. 2011). However, longitudinal studies have demonstrated mixed results when examining CD4 nadir in relation to global cognition (Heaton et al. 2015; Naveed et al. 2022; Ellis et al. 2022). To address this gap, the current study investigated neurocognitive changes by specific neurocognitive domains, as well as globally. These domains were identified empirically and included speed and executive function (S/EF), visuospatial memory (VM), and verbal fluency (VF). Participants had largely well-treated HIV and were followed for an average of 12 years and up to 27 years after CD4 nadir.

The trajectory of global cognition showed a trend towards a statistically significant association with CD4 nadir. However, domain-specific examinations revealed differing associations between the three neurocognitive domains. CD4 nadir was not associated with trajectories of either S/EF or VM, nor was it associated with the overall level of performance in these domains. By comparison, CD4 nadir was significantly associated with VF trajectory, such that those with a lower CD4 nadir (< 200) demonstrated improvement in VF performance over time. They began with a weakness in VF but ultimately eclipsed those with a higher CD4 nadir (> 200), who were stable in that domain. However, when examining reliable change indices (RCIs), which account for factors such as practice effect and regression to the mean, trajectories of VF in these two groups did not differ significantly from one another. Descriptively, improvement was slightly more common and decline slightly less common among those with low nadir. Furthermore, practice effects are typically weaker in patients with neurologic conditions (for a review, see Holm et al. 2022); thus, even practice-related improvements in the low nadir group arguably reflect a favorable outcome. Taken together, findings indicate that CD4 nadir-related cognitive deficits do not worsen significantly over time. Results are mixed as to whether these deficits persist or resolve. The discrepancies between mixed model and RCI results affected multiple cognitive domains and are discussed further below.

The present findings also indicate that the long-term cognitive consequences of low CD4 nadir vary by domain. Heterogeneity across domain-specific trajectories is consistent with prior research in PLWH. Namely, heterogeneity in neurocognitive domain changes has been observed following cART initiation. One study found that PLWH initiating cART treatment demonstrate improved trajectories in VF, psychomotor speed, and executive function, but not episodic memory (Cohen et al. 2001). Cohort studies comparing the cART and pre-cART eras show improvements in VF, visuoconstruction, and attention since the advent of cART, but declines in learning and complex attention (Cysique et al. 2004). In another longitudinal study of women with HIV, verbal fluency, speed, and executive function were stable overall, whereas learning, memory, attention/working memory, and motor function declined (Rubin et al. 2017). Our current findings, together with the aforementioned studies, suggest that trajectories of VF in the setting of effective treatment are more favorable than trajectories of memory. This variability across domains highlights the importance of examining domain-specific cognitive trajectories, as lumping all domains together may obscure important associations, and may explain prior mixed findings when CD4 nadir was examined in relation to global cognitive change (Heaton et al. 2015; Naveed et al. 2022; Ellis et al. 2022).

Immune reconstitution following immunocompromise could be a potential factor contributing to neurocognitive improvement in those who have experienced low CD4 nadir. In line with this hypothesis, one study observed a decreased rate of cerebral atrophy in PLWH who had improving CD4 count trajectories (Pfefferbaum et al. 2014). To explore whether immune reconstitution might explain VF improvement among individuals with low CD4 nadir, we investigated CD4 count trajectories in relation to CD4 nadir and to neurocognitive changes. A significantly larger percentage of low nadir participants demonstrated clinical improvement in CD4 count over time (i.e., from < 500 to > 500 cells/μl). Furthermore, clinically significant improvement in VF was more common in patients with improving CD4 trajectories than in those with stable or declining VF trajectories (21% vs. 8%), but this difference was not statistically significant. These analyses provide some information for a possible mediating variable behind our primary findings but should be interpreted with caution given the lack of statistical significance in the latter analysis. Future research should investigate other potential mechanisms, which were outside the scope of this study.

In addition to the primary findings, baseline global cognitive status was significantly associated with trajectories of S/EF and VM, and with the overall level of performance in all three domains. Whether or not reliable change indices were used, participants with impaired global baseline cognition showed improvement in S/EF at a significantly higher rate than those with intact baseline global cognition. Findings were more mixed in the VM domain but also consistently demonstrated a more favorable trajectory among those with impaired versus intact baseline global cognition. These findings highlight the importance of considering initial global cognitive status when examining longitudinal change, as combining such groups can obscure their unique trajectories. The speed/executive function findings demonstrate the tendency of cognitive impairment to lessen, though not entirely resolve, over time in well-treated PLWH. This finding is in line with studies that found relative stability of cognition over time (Cole et al. 2007; Sacktor et al. 2016; Aung et al. 2023). By comparison, memory results are more consistent with other findings that demonstrate longitudinal decline among PLWH (Grant et al. 2014; Goodkin et al. 2017; Rourke et al. 2021).

Across domains, RCI findings painted a less favorable picture of cognitive trajectories than unadjusted analyses. To some degree, this is to be expected given that the Maassen (2004) RCI method accounts for factors such as practice effects and regression to the mean, which would have inflated performance at follow-up assessments (Maassen 2004; Hinton-Bayre 2010). The use of the Maassen RCI method is indeed a strength of the present study. The norms used to generate RCIs (Woods et al. 2006) also offered several advantages, such as a similar test battery, prior validation in PLWH, and a longer inter-test interval (one year) than other test-retest studies (e.g., one week; Hammers et al. 2021). However, there are also limitations of the RCI approach and the chosen norms. First, the present study had even longer inter-test intervals, with an average of five years, and the Woods et al. (2006) norms do not account for the normal age-related decline that would be expected over a five-year span. As a result, the current RCI findings might depict cognitive change in an overly negative manner. Another weakness of the RCI analyses is their reduced statistical power due to 1) using categorical rather than continuous cognitive trajectories, and 2) including each participant only once instead of two to four times. Given these limitations, a preferred approach would be to include an uninfected control sample assessed over the same time frame. Such a sample is presently being recruited and evaluated at the CNHC and will be incorporated into future evaluations of longitudinal cognitive change once sufficient follow-up data are available.

The present study possesses several other strengths, most notably our domain-specific neurocognitive assessment, which contrasts with the global method utilized in previous studies. This allows our study to provide a more detailed understanding of specific neurocognitive pathways that may be affected by immunocompromise. Simultaneously, empirical dimension reduction of the neurocognitive measures via PCA limits multiple comparisons and ensures the domains assessed reflect distinct constructs. The longitudinal study design and long duration (up to 9 years of follow-up, up to 27 years after CD4 nadir) allows examination of long-term cognitive trajectories of low CD4 nadir, which have been understudied in the largely cross-sectional extant literature. The longitudinal design and the use of linear mixed effects modeling also enable each participant to serve as their own control, which reduces the impact of confounding factors and enhances the external validity of the findings by allowing a less restrictive sample. Additionally, linear mixed effects models use maximum likelihood estimation, which allows for variability and specificity in follow-up durations and limits biases associated with missing data.

Study limitations include the lack of verbal memory measures in the neurocognitive battery; however, HAND is a bilateral brain disorder (Chiang et al. 2007; Thurnher and Donovan Post 2008), with comparable deficits in the verbal and visuospatial modalities of episodic memory (Reger et al. 2002). Thus, we adequately captured episodic memory with the visuospatial modality. Additionally, the retrospective approach in obtaining CD4 nadir counts and dates may have introduced some inaccuracy in these values and prevented us from prospectively examining trajectories immediately following CD4 nadir, as baseline visits were 6 to 7 years after CD4 nadir on average. The slightly lower viral load in patients with versus without follow-up visits raises the possibility of selection/retention bias, as the present results may reflect a sample that is somewhat healthier or more engaged in care; however, this difference was small and did not reach the threshold of statistical significance. Finally, the observational nature of the study design inevitably prevents any causal implications of our findings. Nonetheless, findings provide valuable information regarding observed cognitive trajectories in those with low versus high CD4 nadir.

In sum, the present findings enhance our understanding of long-term neurocognitive trajectories following immunocompromise in PLWH. Varying results across neurocognitive domains suggests that separate neurocognitive pathways may be affected differentially following immunocompromise. Most importantly, these results provide reassurance to patients who have low CD4 nadir counts and are fearful of associated neurocognitive disease progression, as the findings suggest a positive long-term prognostic outlook after acute immunocompromise. Findings similarly suggest a positive long-term prognostic outlook in those who initially present with impairment in speed and executive function, which tends to lessen over time. The main unfavorable finding pertains to the trajectories of visuospatial memory, particularly in those who initially present with intact cognition. This apparent decline might relate to aging and/or normative limitations discussed above. Long-term memory trajectories of older adult PLWH are a topic of further study.

Future studies should incorporate neuroimaging to assess whether domain-specific changes in immune status and neurocognition relate to regional changes in brain structure and function. For example, verbal fluency changes might correspond with inferior frontal and lateral temporal alterations (Heim et al. 2008; Bernal et al. 2015), whereas psychomotor speed and set-shifting changes might relate to alterations in dorsolateral prefrontal cortex and frontostriatal networks (Cunnington et al. 2005; Simard et al. 2011). Further research should also explore other potential mechanisms of cognitive improvement, such as changes in pro-inflammatory cytokines or other immune and viral markers, as well as the impact of cART toxicity, aging, and comorbid conditions. Together, such investigations will further our understanding of long-term trajectories of HAND.

## Supplementary Information

Below is the link to the electronic supplementary material.

Supplementary file1 (PDF 99 KB)

Supplementary file2 (PDF 48 KB)

Supplementary file3 (PDF 80 KB)

## Data Availability

Data will be made available on request.
