# Supplementary material for: CD4 nadir and neurocognitive trajectories in people living with HIV
Source: J Neurovirol. 2024 Jun 10;30(4):423–33. doi: 10.1007/s13365-024-01217-8 (PMC11512832; doi:10.1007/s13365-024-01217-8)
Supplement: Supplementary file 1 — Supplementary file1 (PDF 99 KB) [file 13365_2024_1217_MOESM1_ESM.pdf]

	All Participants	Single-Visit Participants	Multi-Visit Participants	p
**N**	301	169	132	-
**Age[a]**	51.6 (7.6)	51.5 (9.1)	51.8 (7.6)	.725
**Education (years)[a]**	11.9 (2.2)	11.9 (2.2)	11.8 (2.3)	.580
**Gender[b]**	62% Men<br>38% Women	65% Men<br>35% Women	59% Men<br>41% Women	.341
**Race[b]**	88% Black<br>7.3% White<br>3.0% Hispanic<br>1.3% Other	85% Black<br>10% White<br>3.6% Hispanic<br>1.2% Other	92% Black<br>3.8% White<br>2.3% Hispanic<br>1.5% Other	.157
**Nadir CD4 (<200 cells/µl)[b]**	40%	42%	38%	.547
**Latest HIV Viral Load (<200 copies/ml)[b]**	86%	82%	91%	.054
**On cART[b]**	98%	99%	98%	.657
**Latest CD4 Count (cells/µl)[a]**	675 (349)	666 (383)	686 (302)	.622
**Years Seropositive[a]**	18.9 (7.7)	19.3 (8.1)	18.3 (7.3)	.235
**Years on cART[a]**	16.2 (7.9)	16.5 (8.4)	15.7 (7.3)	.400
**Years Seropositive Before Starting cART[a]**	2.9 (4.8)	2.8 (4.7)	3.1 (5.0)	.560
**Years Since Nadir Date[a]**	6.5 (4.1)	6.4 (4.3)	6.5 (3.8)	.754
**Impaired Global Cognitive Status[b]**	54%	54%	52%	.725

[a] Numerical variable. Values represent M (SD). Significance obtained from ANOVA.

[b] Categorical variable. Significance obtained from Fisher’s exact test.
