# Supplementary material for: CD4 nadir and neurocognitive trajectories in people living with HIV
Source: J Neurovirol. 2024 Jun 10;30(4):423–33. doi: 10.1007/s13365-024-01217-8 (PMC11512832; doi:10.1007/s13365-024-01217-8)
Supplement: Supplementary file 2 — Supplementary file2 (PDF 48 KB) [file 13365_2024_1217_MOESM2_ESM.pdf]

	**Component 1: Speed/ Executive Function**	**Component 2: Visuospatial Memory**	**Component 3: Verbal Fluency**
**Letter Fluency**	-	-	0.79
**Category Fluency**	-	-	0.88
**Trails A**	0.75	-	-
**Trails B**	0.64	-	-
**Grooved Pegboard – Dominant**	0.83	-	-
**Grooved Pegboard – Non-Dominant**	0.87	-	-
**BVMT-R Immediate**	-	0.86	-
**BVMT-R Delayed**	-	0.86	-
**BVMT-R Recognition**	-	0.74	-

Loadings less than 0.4 were omitted for clarity.
