# Supplementary material for: CD4 nadir and neurocognitive trajectories in people living with HIV
Source: J Neurovirol. 2024 Jun 10;30(4):423–33. doi: 10.1007/s13365-024-01217-8 (PMC11512832; doi:10.1007/s13365-024-01217-8)
Supplement: Supplementary file 3 — Supplementary file3 (PDF 80 KB) [file 13365_2024_1217_MOESM3_ESM.pdf]

**Number of Clusters**	**Schwarz's Bayesian Criterion (BIC)**	**BIC Change**	**Ratio of BIC Changes**	**Ratio of Distance Measures**	**Silhouette Measure of Cohesion and Separation**
**1**	646.06	-	-	-	-
**2**	**513.63**	**-132.43**	**1.00**	**2.26**	**0.44**
**3**	474.10	-39.53	0.30	1.50	0.35
**4**	459.14	-14.96	0.11	1.32	0.38
**5**	456.07	-3.07	0.02	1.60	0.37
**6**	467.01	10.94	-0.08	1.15	0.37
**7**	481.04	14.03	-0.11	1.13	0.35
**8**	497.42	16.38	-0.12	1.17	0.35
**9**	516.40	18.98	-0.14	1.25	0.36
**10**	538.39	21.10	-0.17	1.09	0.37
